# Supplementary material for: Comparative Analysis and Phylogenetic Insights of Cas14-Homology Proteins in Bacteria and Archaea
Source: Genes (Basel). 2023 Oct 6;14(10):1911. doi: 10.3390/genes14101911 (PMC10606334; doi:10.3390/genes14101911)
Supplement: Supplementary file 1 [file genes-14-01911-s001.zip › Suplementry_Data/Suplementry Figure 2.pdf]

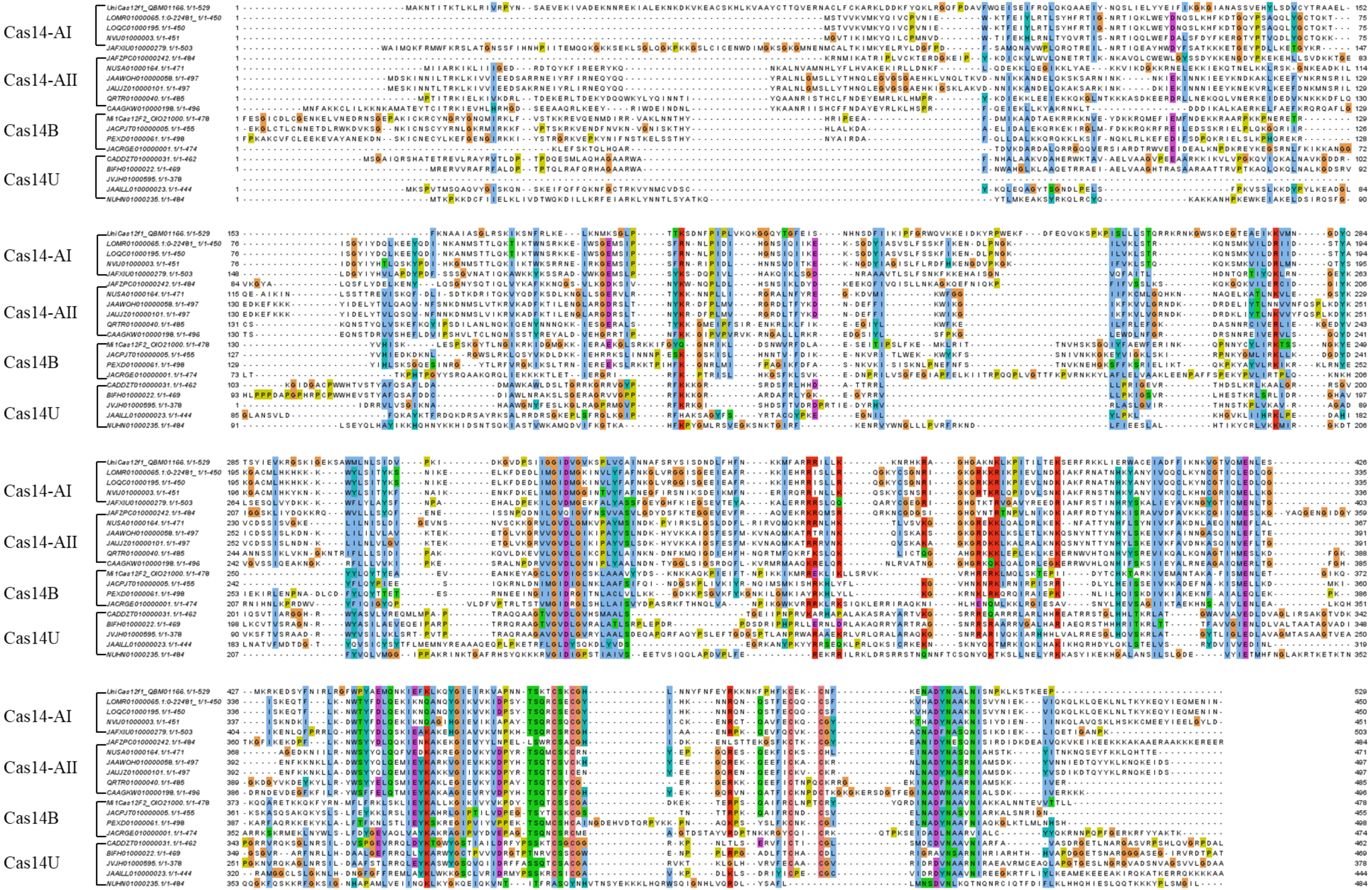
Cas14-AI
Cas14-AII
Cas14B
Cas14U
