# Supplementary material for: Comparative Analysis and Phylogenetic Insights of Cas14-Homology Proteins in Bacteria and Archaea
Source: Genes (Basel). 2023 Oct 6;14(10):1911. doi: 10.3390/genes14101911 (PMC10606334; doi:10.3390/genes14101911)

A)

Average of mined-Cas14 sequence Identity (overall : 18±15%)

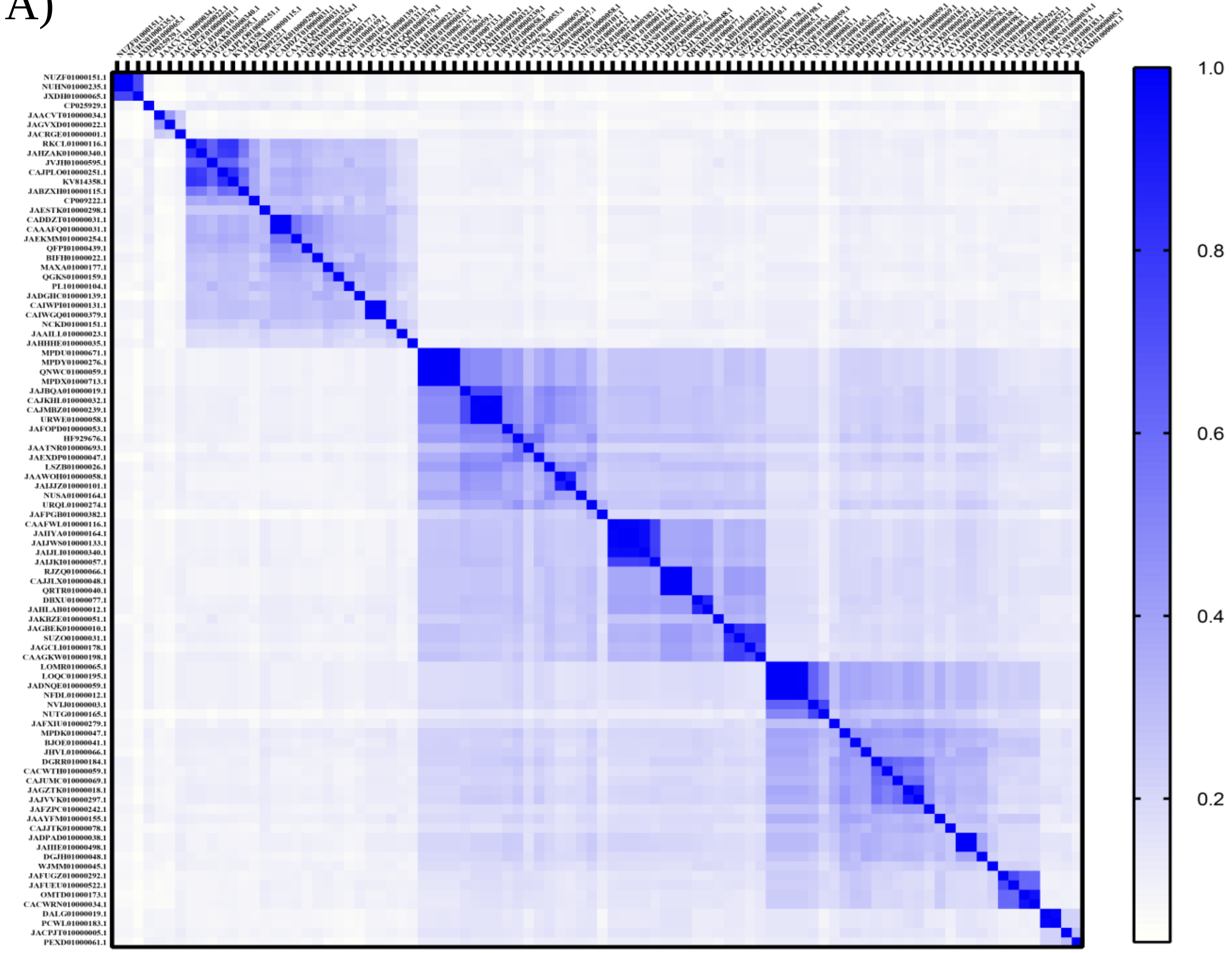

B)

Average of mined-Cas14U sequence Identity (overall : 27±22%)

Average of mined-Cas14A sequence Identity (overall : 26±16%)

Average of mined-Cas14B sequence Identity (overall : 36±33%)

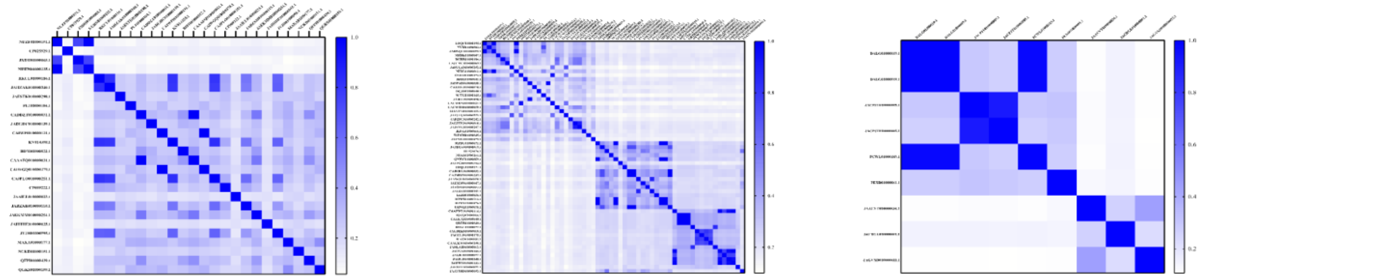

Supplement: Supplementary file 1 [file genes-14-01911-s001.zip › Suplementry_Data/Suplementry Figure 4.pdf]
